# Altitude, Exercise, and Skeletal Muscle Angio-Adaptive Responses to Hypoxia: A Complex Story

**DOI:** 10.3389/fphys.2021.735557

**Published:** 2021-09-06

**Authors:** Pierre Lemieux, Olivier Birot

**Affiliations:** Muscle Health Research Centre, School of Kinesiology and Health Science, York University, Toronto, ON, Canada

**Keywords:** skeletal muscle, capillary, angiogenesis, VEGF-A, thrombospondin, hypoxia, altitude, exercise

## Abstract

Hypoxia, defined as a reduced oxygen availability, can be observed in many tissues in response to various physiological and pathological conditions. As a hallmark of the altitude environment, ambient hypoxia results from a drop in the oxygen pressure in the atmosphere with elevation. A hypoxic stress can also occur at the cellular level when the oxygen supply through the local microcirculation cannot match the cells’ metabolic needs. This has been suggested in contracting skeletal myofibers during physical exercise. Regardless of its origin, ambient or exercise-induced, muscle hypoxia triggers complex angio-adaptive responses in the skeletal muscle tissue. These can result in the expression of a plethora of angio-adaptive molecules, ultimately leading to the growth, stabilization, or regression of muscle capillaries. This remarkable plasticity of the capillary network is referred to as angio-adaptation. It can alter the capillary-to-myofiber interface, which represent an important determinant of skeletal muscle function. These angio-adaptive molecules can also be released in the circulation as myokines to act on distant tissues. This review addresses the respective and combined potency of ambient hypoxia and exercise to generate a cellular hypoxic stress in skeletal muscle. The major skeletal muscle angio-adaptive responses to hypoxia so far described in this context will be discussed, including existing controversies in the field. Finally, this review will highlight the molecular complexity of the skeletal muscle angio-adaptive response to hypoxia and identify current gaps of knowledges in this field of exercise and environmental physiology.

## Introduction

**FIGURE 1:**
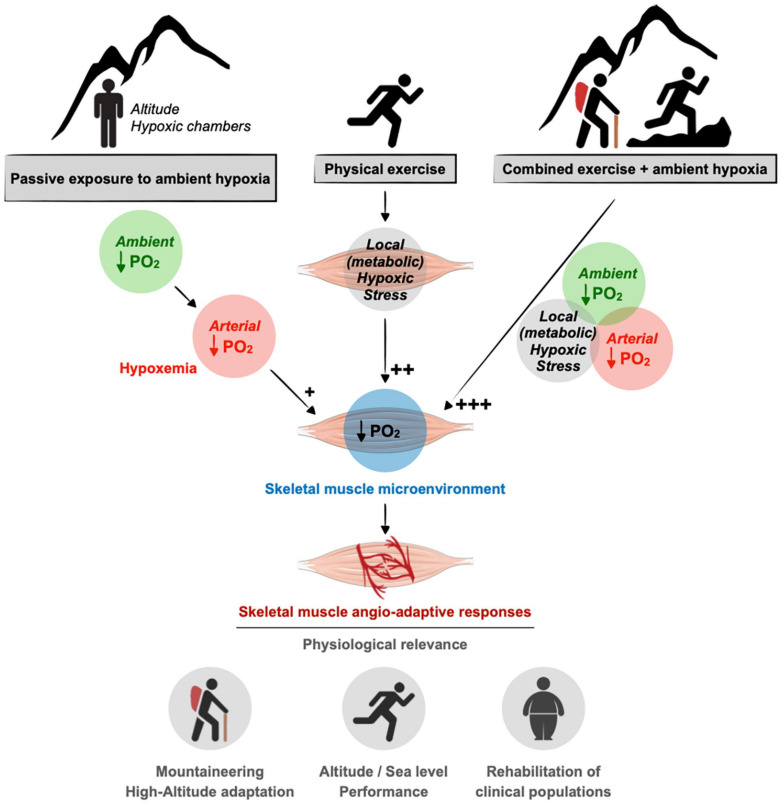
Contribution of ambient hypoxia and exercise to induce a hypoxic stress in skeletal muscle. A drop in the partial pressure of oxygen (PO_2_) in the muscle tissue can result from a decrease in ambient PO_2_ (e.g., altitude) as well as an unbalanced between blood supply to the muscle cells and their increase metabolic needs during exercise. In response to muscle hypoxia angio-adaptive responses will take place to maintain an optimal oxygen supply to myofibers and to preserve muscle function. This is of particular relevance in the context of mountaineering activity as well as exercise training strategies using ambient hypoxia for athletes or clinical populations. As discussed in the text, the impact of ambient hypoxia on skeletal muscle PO_2_ seems rather modest (+) compared to the impact of exercise in generating a local hypoxic stress (++). The combination of ambient hypoxia exposure and exercise might exacerbate this hypoxic stress (+++).

Our review aims to revisit the complexity of the skeletal muscle angio-adaptive response to hypoxia, particularly when combining exposure to ambient hypoxia and exercise-induced tissue hypoxia. Indeed, in the context of high-altitude expeditions, mountaineers usually engage into prolonged periods of intense physical activity over several weeks or months (West, 2006, 2012). In the context of sport performance at sea level, hypoxia training has become a complex and very specialized area of research (Millet et al., 2010; Lundby et al., 2012; Girard and Chalabi, 2013; Girard and Pluim, 2013; Girard et al., 2013). Finally, the use of exercise training under hypoxia has recently emerged as a new and promising therapeutic avenue to improve some metabolic and cardiovascular conditions (obesity, type-2 diabetes, hypertension) as well as for the training of elderly subjects (Verges et al., 2015; Millet et al., 2016; Figure 1).

Skeletal muscles represent one of our largest tissues, accounting for about 40% of human body weight. Skeletal muscles adapt to environmental, physiological, and pathological conditions with a remarkable plasticity. This can include changes in muscle mass, in the size of myofibers and their metabolic and contractile phenotype, as well as changes in muscle capillarization (Booth and Thomason, 1991; Hudlicka et al., 1992; Hudlicka, 2011).

Since August Krogh’s pioneering work about a century ago (Krogh, 1919a,b,c), our understanding of the regulation of muscle blood flow and oxygen delivery to muscle cells has considerably evolved and was recently revisited in great review articles (Angleys and Østergaard, 2020; Poole et al., 2020, 2021; Kissane et al., 2021). The oxygen cascade from skeletal muscle arterioles to capillaries, interstitial tissue, sarcolemma, and mitochondria can be influenced at several levels: The vasomotricity of upstream arterioles and the subsequent regulation of capillary blood flow; the content and velocity of red blood cells; the hemoglobin and myoglobin concentrations; the tortuosity and number of capillaries; and the surface area of myofibers.

The capillary-to-myofiber interface plays a crucial role for muscle function. Indeed, it represents the site of exchange for oxygen, nutrients, metabolic heat and waste between the blood and the myofibers. The density of capillaries within a given area of muscle tissue will greatly contribute to matching the delivery of oxygen and nutrients with the myofibers’ metabolic needs, particularly during contractile activity (Hudlicka et al., 1987; Hoppeler and Kayar, 1988; Mathieu-Costello, 1994; Hudlicka, 2011). The capillary network can therefore be considered as a key determinant of skeletal muscle function and several studies have reported strong correlations between the level of muscle capillarization and mitochondria volume density, muscle oxidative capacity, and oxygen consumption (Hoppeler et al., 1987; Hudlicka et al., 1987; Hoppeler and Kayar, 1988; Poole and Mathieu-Costello, 1996; Howlett et al., 2003). For instance, Howlett et al. (2003) reported a strong correlation between skeletal muscle capillary density and muscle oxygen conductance in rats selectively bred for running endurance (Howlett et al., 2003).

## Skeletal Muscle Capillarization and the Concept of Muscle Angio-Adaptation

The capillary density in a muscle section can vary in response to various environmental, physiological, or pathological conditions. An increase in muscle capillary density is usually observed in human subjects and animal models in response to prolonged endurance training or high-altitude sojourn (Hudlicka et al., 1992; Breen et al., 2008; Hudlicka, 2011). Conversely, skeletal muscle capillary rarefaction has been described in response to physical deconditioning as well as in the context of some pathologies such as chronic obstructive pulmonary disease, chronic heart failure, or diabetes (Roudier et al., 2010; Gouzi et al., 2013; Olfert et al., 2015; Aiken et al., 2019).

Skeletal muscle angio-adaptation refers to the complex and dynamic processes of capillary formation, stabilization, or regression in response to acute and chronic physiological or pathological conditions. These processes are regulated at the molecular level by a plethora of pro- and anti-angiogenic molecules (Hoppeler, 1999; Breen et al., 2008; Egginton, 2009; Olfert and Birot, 2011; Egginton and Birot, 2014; Olfert et al., 2015). At the cellular level, endothelial cells proliferate, migrate, and assemble to form new capillaries in the context of angiogenesis, or conversely, undergo apoptosis during capillary regression. Myofibers can represent an important source of production and release of angio-regulatory molecules such as the well-described pro-angiogenic Vascular Endothelial Growth Factor-A (VEGF-A).

Importantly, changes in capillary density can also be a direct consequence of alterations in the size of myofibers (hypertrophy or atrophy) without any true capillary loss or formation. Also, no change in capillary density is not necessarily synonymous of an absence of angio-adaptive activity. For example, during myofiber hypertrophy, a formation of new capillaries might occur simply to prevent a decrease in the capillary density that would result from the increase in the myofibers surface area. It is also important to note that some angio-adaptive molecules, such as the pro-angiogenic VEGF-A, are not only required for the growth of capillaries but also to maintain existing ones. Evidence suggests an autocrine expression of VEGF-A being important for endothelial cell survival and vascular homeostasis (Lee et al., 2007; Domigan et al., 2015). Finally, the skeletal muscle could also be seen as an endocrine organ releasing “angio-adaptive myokines” in circulation, potentially affecting distant tissues.

## Skeletal Muscle Hypoxia: Impact of Altitude and Exercise

Hypoxia, defined as a lack of oxygen supply to a given tissue, is a powerful pro-angiogenic stimulus for various cell types and tissues including skeletal muscle (Hoppeler and Vogt, 2001; Semenza, 2001; Breen et al., 2008; Fraisl et al., 2009; Favier et al., 2015). Ambient hypoxia is a hallmark of the altitude environment and results from a drop in the oxygen pressure in the atmosphere with elevation. However, the impact of ambient hypoxia *per se* on skeletal muscle angio-adaptive responses and capillarization still remains a source of scientific debate. A hypoxic stress can also be generated locally at the muscle tissue level in response to intense exercise if oxygen delivery cannot match the metabolic needs of contracting myofibers (Figure 1).

Several studies have aimed at determining the oxygen partial pressure (PO_2_) cascade in human and animal models using different techniques such as proton magnetic resonance spectroscopy, surface electrodes, microcatheter, and more recently phosphorescence quenching. The intramuscular PO_2_ at rest is estimated around 27 mmHg with some variations (10-34 mmHg) among studies (different species, muscles, and techniques), below the microvascular PO_2_ but well above the intra-myocyte PO_2_ (1-3 mmHg) (Prewitt and Johnson, 1976; Boegehold and Johnson, 1988; Richardson et al., 1995, 2006; Richmond et al., 1997, 1999; Hutter et al., 1999; Molé et al., 1999; Lombard et al., 2000; Richardson, 2000; Kindig et al., 2003; Johnson et al., 2005; Liu et al., 2012; Hirai et al., 2018, 2019; Colburn et al., 2020; Poole et al., 2020, 2021).

A few of these studies have investigated the impact of ambient hypoxia on skeletal muscle PO_2_. A modest reduction in PO_2_ (from 34 mmHg under normoxia to 23 mmHg under hypoxia) was observed in human quadriceps muscle following an exposure to an inspired O_2_ fraction of 10% (F_i_O_2_ 0.10), equivalent to an altitude of about 5,800 m (Richardson et al., 2006). In another study, the interstitial PO_2_ in rat cremaster muscles was reduced from 26 to about 10 mmHg following one minute exposure to an inspired O_2_ fraction of 7% (F_i_O_2_ 0.07), equivalent to an altitude of about 8,300 m (Johnson et al., 2005). However, the physiological relevance of this conditioning (1 min of exposure at F_i_O_2_ 0.07) can be questioned. These few studies suggest that ambient hypoxia exposure might not alter muscle PO_2_ to a large extent. Conversely, the impact of physical exercise on the muscle PO_2_ seems much more important. One bout of exercise, even at moderate intensity (50% maximal leg O_2_ uptake) was shown to decrease muscle PO_2_ to values around 3-5 mmHg (Richardson et al., 1995, 2006; Molé et al., 1999; Angleys and Østergaard, 2020). This exercise-induced hypoxic stress could be further exacerbated (muscle PO_2_ down to 2 mmHg) when exercise was performed in a hypoxic environment (Richardson et al., 2006).

At a molecular level, the Hypoxia Inducible Factor-1 (HIF-1) is a transcription factor widely recognized as a hallmark of the hypoxia signaling pathway (Semenza and Wang, 1992; Semenza, 2001). HIF-1 is a heterodimeric complex formed of two subunits alpha and beta. Whereas the expression of the beta subunit remains stable under both normoxic and hypoxic conditions, the alpha subunit (HIF-1α) confers on HIF-1 most of its regulation. Under normoxia, the HIF-1α protein is constantly synthesized in the cytoplasm, and rapidly undergoes proteosomal degradation within a few minutes. This involves HIF-1α hydroxylation on certain proline residues by HIF prolyl hydroxylases (PHD1-3) (Ke and Costa, 2006; Lindholm et al., 2014). If the PO_2_ drops enough to generate a hypoxic stress at the cellular level, the function of PHD1-3 is inhibited, HIF-1α is stabilized and can translocate into the nucleus to dimerize with the beta subunit. HIF-1 binds to hypoxia responsive elements (HRE) in the promoter regions of target genes to regulate their transcription. Both the erythropoietin (EPO) and pro-angiogenic VEGF-A genes possess HRE sites in their promoters (Semenza, 2001).

Whether ambient hypoxia exposure results in an increased expression of HIF-1α protein in skeletal muscle remains largely understudied. Stroka et al. (2001) have observed a strong HIF-1α protein expression level in mouse skeletal muscle even under normoxic conditions (Stroka et al., 2001). In the same study, 1 h exposure to extreme normobaric hypoxia (F_i_O_2_ 0.06, equivalent to 9,100 m) did not seem to increase the expression level much further. As noted previously, the physiological relevance of conditionings combining very short exposures (1 min to 1 h) and extreme hypoxia levels (F_i_O_2_ 0.06-0.07) are questionable (Stroka et al., 2001; Johnson et al., 2005).

Intense physical exercise can be at the origin of a local hypoxic stress in the muscle tissue. In an elegant study, Ameln et al. (2005) quantified HIF-1α protein expression in response to one single bout of moderate intensity exercise in human vastus lateralis muscle biopsies (Ameln et al., 2005). HIF-1α protein levels were increased immediately after exercise and remained elevated for up to 6 h post-exercise. An increased nuclear staining for HIF-1α was also observed as well as an increased DNA binding to HRE binding sites. mRNA levels for HIF-1 target genes EPO and VEGF-A were also higher post-exercise. These results suggest that HIF-1 expression and activity were both increased post-exercise in human skeletal muscle.

An increase in HIF-1α protein expression could result from the inhibition of HIF-1α protein degradation but also from an increased expression of HIF-1α mRNA and protein translation. Vogt et al. (2001) have measured HIF-1α mRNA in human vastus lateralis muscle biopsies before and after an endurance training program conducted at low or high intensity either under normoxia or normobaric hypoxia (simulated altitude of 3,850 m) (Vogt et al., 2001). HIF-1α mRNA expression was increased after hypoxia training regardless of the training intensity whereas training under normoxia had no effect. Interestingly, VEGF-A mRNA levels were also measured and found to be increased only in the high-intensity hypoxia training group but not under low exercise training conditions. This could reveal a synergetic effect of combining ambient hypoxia and exercise-induced local hypoxia. Here, biopsies were performed at least 24 h post-exercise to assess the effect of prolonged training on HIF-1α basal levels. In line with this study, Lundby et al. (2006) have evaluated the impact of prolonged exercise training on HIF-1α acute response to one single bout of exercise (Lundby et al., 2006). HIF-1α mRNA levels were increased in vastus lateralis biopsies at 6 h post-exercise only in the untrained group. Whether exercise training could blunt the acute HIF-1α response to one single bout of exercise was then questioned by Lindholm et al. (2014), Lindholm and Rundqvist (2016) who showed that several inhibitors of HIF-1α expression and HIF-1 activity were increased in trained muscles. The analysis of trained muscle biopsies indeed revealed higher expression levels of prolyl hydroxylases (PHD1-3), Factor Inhibiting HIF-1 (FIH) and Sirtuin-6 (SIRT6) (Lindholm et al., 2014). By catalyzing hydroxylation on specific prolyl residues, PHD1-3 target HIF-1α for proteasomal degradation. FIH, a HIF-1α-specific asparagine hydroxylase, can inhibit HIF-1 transactivation. SIRT6, a histone deacetylase, can act as an epigenetic co-repressor of HIF-1. Another explanation to the results from Lundby et al. (2006) could be an increased level of muscle capillarization post-training. Angiogenesis is a well-described tissular adaptation of the skeletal muscle tissue to endurance training. This would result in a better capillary-to-myofiber interface and oxygen delivery to contracting myofibers, and as such, a reduced exercise-induced hypoxic stress in trained muscles.

In addition to inducing a local cellular hypoxic stress, exercise combines other pro-angiogenic stimuli such as increased shear stress on endothelial cells due to enhanced muscle blood flow, mechanical tissue stretch, and oxidative stress (Hudlicka et al., 1992; Milkiewicz et al., 2001; Breen et al., 2008; Egginton, 2009; Hoier et al., 2012; Egginton and Birot, 2014; Hellsten and Hoier, 2014; Haas and Nwadozi, 2015; Olfert et al., 2015). The increase in muscle blood flow results from active vasodilation, and the resultant increase in shear stress represents a well-described pro-angiogenic stimulus for skeletal muscle endothelial cells, mediating the production of angio-adaptive molecules such as nitric oxide, metalloproteinases, and VEGF-A (Milkiewicz et al., 2001, 2011; Egginton, 2009, 2011; Hudlicka and Brown, 2009; Hellsten and Hoier, 2014; Haas and Nwadozi, 2015). If local cellular hypoxia and shear stress are often presented side by side as exercise-induced pro-angiogenic stimuli, passive exposure to ambient hypoxia also stimulates vasodilation and increases blood flow in skeletal muscle (Casey and Joyner, 2011, 2012; Joyner and Casey, 2014; Dinenno, 2016). Therefore, ambient hypoxia could then represent an upstream stimulus of muscle shear stress. The degree of vasodilation observed seems linked to the degree of ambient hypoxia, at least for acute exposures. Interestingly, the combination of ambient hypoxia exposure and exercise seems to synergistically induce greater vasodilation and muscle blood flow than what could be expected by simply adding their respective contributions (Casey and Joyner, 2011, 2012; Joyner and Casey, 2014). Finally, to add more complexity, HIF-1α expression can also be stimulated under normoxic conditions in skeletal muscle in response to increased shear stress or mechanical tissue stretch (Milkiewicz et al., 2007).

## Altitude, Exercise, and Skeletal Muscle Hypoxia: Key Points

• Exercise is a more powerful stimulus than ambient hypoxia to decrease muscle PO_2_.
• Combining ambient hypoxia and exercise might further decrease muscle PO_2_.
• Hypoxia Inducible Factor-1 is a well-established hallmark of hypoxia signaling.
• Skeletal muscle angio-adaptive activity can locally change the level of muscle capillarization and can also release angio-adaptive myokines in the circulation for distant effects.

## Increased Muscle Capillarization in Response to Exercise Training and Ambient Hypoxia

We previously discussed how increasing the capillary-to-myofiber interface could be beneficial for maintaining or improving muscle function when oxygen delivery becomes a challenge. The capillary density (CD), which represents the number of capillaries per surface unit of tissue, is often used to assess the level of muscle capillarization (Andersen, 1975; Hudlicka et al., 1992). The CD can however be influenced both by changes in the number of capillaries and alterations in the size of myofibers. Changes in CD might therefore not always be representative of angiogenesis or capillary regression. Conversely, the capillary-to-fiber ratio (C/F) represents one of the best histological parameters to truly appreciate capillaries formation or rarefaction.

Exercise training is a powerful and well-established pro-angiogenic stimulus for the skeletal muscle tissue (Andersen, 1975; Andersen and Henriksson, 1977; Hudlicka et al., 1992; Egginton, 2009). Human and animal studies have shown that one single bout of exercise leads to the production and release of several angio-adaptive molecules both in the muscle microenvironment, for example to stimulate skeletal muscle endothelial cells (Roudier et al., 2012; Aiken et al., 2016), and in the circulation as myokines (Hudlicka et al., 1992; Vogt et al., 2001; Egginton, 2009; Olfert and Birot, 2011; Hoier et al., 2012; Egginton and Birot, 2014). During prolonged exercise training, the chronic repetition of these exercise-induced angiogenic responses can ultimately lead to the formation of new capillaries. This increase in muscle capillarization will usually be reflected by higher C/F and CD in trained muscles (Andersen, 1975; Andersen and Henriksson, 1977; Hudlicka et al., 1992; Egginton, 2009). As previously mentioned, the exercise stimulus combines several stressors such as local tissue hypoxia, increased shear stress, tissue stretch, and oxidative stress. The exact contribution of hypoxia *per se* during exercise remains difficult to study and still unclear. Conversely to regular exercise, muscle hypokinesia or deconditioning can lead to capillary regression (Fujino et al., 2005; Egginton, 2009; Malek et al., 2010; Roudier et al., 2010; Olfert and Birot, 2011).

The effect of passive exposure to ambient hypoxia on skeletal muscle capillarization is less clear than the impact of exercise training. A well-established consensus of the literature is that prolonged exposure to field or simulated high altitude hypoxia results in improved skeletal muscle capillarization and increased capillary density. This alteration in capillary density seems however mainly due to the atrophy of myofibers rather than a true angiogenic response. Several studies have indeed reported an increase in CD concomitantly to a decrease in the myofiber surface area, with no change in the C/F ratio (Oelz et al., 1986; Green et al., 1989; Hoppeler et al., 1990a,b; MacDougall et al., 1991; Kayser et al., 1996).

**FIGURE 2:**
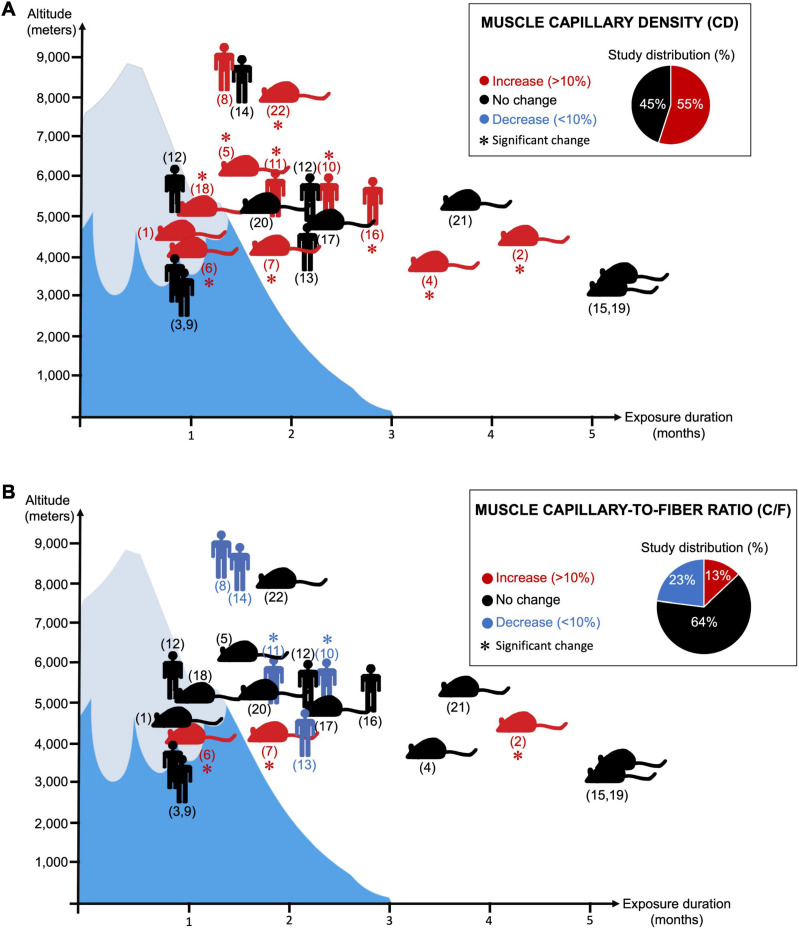
Twenty-two research articles analyzing changes in skeletal muscle capillary density (CD) (Panel **A**) or capillary-to-fiber ratio (C/F) (Panel **B**) in response to prolonged exposure to ambient hypoxia were surveyed. (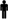) indicates human studies. (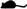) indicates the use of animal models (not restricted to rodents). (^∗^) indicates significant finding. Red coloration indicates an increase in magnitude by 10% or greater. Blue coloration indicates a decrease in magnitude by 10% or greater. Black coloration indicates no change. *Y*- and *X*-axis, respectively, reflect the level of altitude (real or simulated) and the duration exposure. Corresponding references: **(1)** Banchero et al. (1976); **(2)** Banchero et al. (1985); **(3)** Basset et al. (2006); **(4)** Bigard et al. (1991); **(5)** Cassin et al. (1971); **(6)** Deveci et al. (2001); **(7)** Deveci et al. (2002); **(8)** Green et al. (1989); **(9)** Green et al. (1992); **(10)** Hoppeler et al. (1990a); **(11)** Hoppeler et al. (1990b); **(12)** Levett et al. (2012); **(13)** Lundby (2004); **(14)** MacDougall et al. (1991); **(15)** Mathieu-Costello and Agey (1997); **(16)** Mizuno et al. (2008); **(17)** Olfert et al. (2001); **(18)** Panisello et al. (2008); **(19)** Poole and Mathieu-Costello (1989); **(20)** Sillau and Banchero (1977); **(21)** Sillau et al. (1980a); **(22)** van Ekeren et al. (1992).

This should be taken with a certain caution since there are in fact almost as many original studies reporting an increase in muscle CD as studies showing no change (Figure 2A; Banchero et al., 1976; Sillau and Banchero, 1977; Oelz et al., 1986; Poole and Mathieu-Costello, 1989; Hoppeler et al., 1990a,b; Bigard et al., 1991; Green et al., 1992; Kayser et al., 1996; Olfert et al., 2001; Lundby, 2004; Mizuno et al., 2008; Levett et al., 2012). We searched the PubMed database for original research studies that analyzed skeletal muscle CD and C/F in animals or human subjects passively exposed to normobaric or hypobaric hypoxia. Studies combining ambient hypoxia and exercise interventions were included only if they had all the required experimental groups to assess the impact of passive hypoxia exposure independently of the exercise training stimulus. We identified a total of 22 original research studies (Figure 2). Interestingly, 55% reported a significant increase or a trend for an increase in CD by at least 10% whereas 45% showed no change (Figure 2A).

The lack of change in CD could be attributed to an absence of muscle atrophy, and some authors have in fact observed no reduction in myofiber size at high altitude (Green et al., 1992; Lundby, 2004; Levett et al., 2012; D’Hulst et al., 2016). It was then suggested that both the exposure duration and the altitude level could determine whether myofibers would atrophy or not, leading to the notion of hypoxic dose (D’Hulst and Deldicque, 2017; Millet et al., 2017). Some conditionings were indeed performed at moderate altitude (3,000-4,000 m) over 2-3 weeks only, as opposed to longer exposures (8-10 weeks) at higher altitudes (>5,200 m) (Oelz et al., 1986; Green et al., 1989, 1992; Hoppeler et al., 1990a,b; MacDougall et al., 1991; Lundby, 2004; Mizuno et al., 2008; Levett et al., 2012).

It is difficult to identify the source of discrepancy between these studies regarding myofiber size and CD. In rodent studies for example, the age of the animals can be a confounding factor if they are still growing (Banchero, 1985; Snyder et al., 1992). Calorie intake can also influence body and muscle weights. Prolonged mountaineering expeditions at high-altitude can involve logistical constraints for proper nutrition and gastroenteritis disorders are often described (West, 2012; Swenson and Bärtsch, 2013). Hypophagia has been well observed in rodents exposed to prolonged hypoxia, usually requiring the use of pair-fed control animals (Daneshrad et al., 2001, 2003).

Muscle atrophy could also result from reduced physical activity and muscle deconditioning, particularly during prolonged and passive exposure in hypoxic chambers. For example, when maintaining an isocaloric state and matching physical activity levels, Green et al. (1992) did not observe any atrophy in their study. There is however still a lack of consensus around the contribution of physical activity or the notion of a hypoxic dose on muscle atrophy. This can nicely be illustrated by two studies from Mizuno et al. (2008), Levett et al. (2012). Both compared very active versus less active subjects during similar conditions of prolonged exposure (66-75 days) to high altitude (>5,000 m). Whereas the less active participants remained moderately active at the Everest Base Camp (5,250-5,300 m), active subjects were repeating climbing sessions at higher altitudes. In Levett’s study, climbers reached Camp 2 (6,400 m) and some even summited the Mount Everest (8,848 m) (Levett et al., 2012). Mizuno et al. (2008) observed a significant increase in muscle CD (+14%) associated with a reduction in muscle circumferences and myofiber size. Conversely, Levett et al. (2012) did not observe any significant myofiber atrophy or change in CD.

Some authors have also concluded that ambient hypoxia alone might not be sufficient to stimulate skeletal muscle angiogenesis and that a combination of cold and hypoxia stressors might be required (Banchero, 1985; Jackson et al., 1987; Snyder et al., 1992; Hoppeler, 1999). Interestingly, cold *per se* was recently identified as a pro-angiogenic stimulus in rodent skeletal muscle (Sillau et al., 1980b; Egginton, 2002; Deveci and Egginton, 2003).

An increase in muscle CD can also result from the formation of new capillaries via the process of angiogenesis. The capillary-to-fiber ratio (C/F) is often used to assess capillary formation. Some earlier studies in species adapted to high altitude, including deer mice, gooses, finches or pigeons have reported increased C/F values compared to sea level animals (Mathieu-Costello and Agey, 1997; Hepple et al., 1998; Hepple, 2000; Lui et al., 2015; Scott et al., 2015). Yet, as previously mentioned, a general interpretation is that the increase in CD in response prolonged exposure to hypoxia results from tissue remodeling and myofiber atrophy without any angiogenesis. Methodological artifacts in tissue preparation for histology procedures, such as variation in the sarcomere length, were pointed out (Sillau and Banchero, 1977; Sillau et al., 1980b; Banchero, 1985; Banchero et al., 1985; Hoppeler and Kayar, 1988; Poole and Mathieu-Costello, 1989; Hoppeler et al., 1990a,b; Hudlicka et al., 1992; Mathieu-Costello, 1994). The impact of prolonged hypoxia on muscle C/F was however, revisited by Deveci et al. (2001, 2002) in rats passively exposed to 3 or 6 weeks of hypoxia (F_i_O_2_ 0.12, equivalent to 4,400 m). The effect of hypoxia on C/F could vary accordingly to muscle and fiber types as well as exposure duration. After 3 weeks of conditioning, CD and C/F were increased in the diaphragm and soleus muscles without any significant myofiber atrophy, suggesting a true angiogenic response to ambient hypoxia. No indication of angiogenesis was observed in the tibialis anterior and extensor digitorum longus muscles (Deveci et al., 2001). At 6 weeks, an increase in C/F was observed all muscles (Deveci et al., 2002). Interestingly, the analysis of different areas from each muscle also suggested that angiogenesis might occurs predominantly around larger and glycolytic myofibers (Deveci et al., 2001, 2002).

When re-analyzing our 22 original research studies that measured the C/F ratio in animal and human skeletal muscles in response to prolonged exposure to hypoxia, we found that about 64% mentioned no change and only 13% reported a significant increase or a trend for it (Figure 2B). Interestingly, 23% indicated a significant or a trend for a decrease in C/F (Green et al., 1989; Hoppeler et al., 1990a,b; MacDougall et al., 1991; Lundby, 2004). For example, in the study from Hoppeler et al., 1990a,b, the significant decrease in C/F (-10%) is of the same magnitude as the increase in CD (+11%).

Based on the current literature, the understanding of the effect of prolonged exposure to hypoxia on skeletal muscle capillarization is still unclear and difficult to generalize. Responses can vary between species, muscles, and fiber types, and can be different when combining different stressors such as cold or physical activity. The idea of CD increasing because of myofiber atrophy can be seen as an interesting and economic way to improve the capillary-to-myofiber interface without the need of angiogenesis. However, having smaller myofibers might represent a disadvantage when it comes to muscle performance.

Divergent from passive exposure to ambient hypoxia, the utilization of hypoxia in conjunction with exercise has gained popularity as a possible training avenue for improving sea-level exercise performance (Levine, 2002; Levine and Stray-Gundersen, 2006; Millet et al., 2010; Lundby et al., 2012; Chapman, 2013; Girard and Chalabi, 2013; Girard et al., 2013, 2020; Brocherie et al., 2018). As discussed earlier, exercise can generate a local hypoxic stress in the skeletal muscle tissue. It is therefore appealing to consider how the combination of ambient hypoxia and exercise training would affect muscle capillarization.

Several rodent and human studies suggest that skeletal muscle capillarization might improve to a larger extent when exercise training is performed under ambient hypoxia compared to sea level conditions (Terrados et al., 1988; Bigard et al., 1991; van Ekeren et al., 1992; Desplanches et al., 1993, 1996; Olfert et al., 2001; Vogt et al., 2001). To assess the impact of ambient hypoxia on the angiogenic effect of training, some authors trained their animals or subjects at the same relative intensity, for example at a similar percentage of VO_2_ max determined under hypoxia and normoxia, thus expecting similar endurance times. Conversely, some authors utilized training protocols with the same absolute intensity, which then can represent a higher stimulus in hypoxic conditions.

Bigard et al. (1991) observed a greater increase in C/F ratio in the plantaris, extensor digitorum longus and soleus muscles of rats housed and trained in a hypobaric chamber at an altitude equivalent to 4,000 m compared to animals trained under normoxic conditions (Bigard et al., 1991). Yet, in the same study C/F values were not different when comparing sedentary normoxic and hypoxic animals, suggesting that hypoxia alone is insufficient to promote muscle angiogenesis. Similarly, Olfert et al. (2001) trained rats for 8 weeks under ambient hypoxia (FiO_2_ 0.12) or normoxia. The C/F ratio was only increased in muscles from animals trained under hypoxia (Olfert et al., 2001).

Interestingly, Vogt et al. (2001) have also evaluated the effect of exercise intensity during hypoxia training in human subjects. They reported a significant increase in capillarization only in the vastus lateralis muscles of subjects trained at high intensity for 12 weeks under hypoxia (simulated altitude of 3,850 m). Training under normoxia, even at high intensity, did not improve significantly muscle capillarization.

Overall, these laboratory and well-controlled studies indicate a greater angio-adaptive response of the skeletal muscle tissue when exercise training is performed in ambient hypoxia. This reflects into a higher level of muscle capillarization. Yet, it remains unknow whether muscle angio-adaptation occurs faster.

Field studies in humans for prolonged sojourn to altitude focusing on exercise and muscle angiogenic activity have provided mixed results. Mizuno et al. (1990) reported a significant increase in the triceps C/F of competitive cross-country skiers after 2 weeks of training at an altitude of 2,700 m (Mizuno et al., 1990). However, because of the absence of proper control groups (sedentary and trained subjects in normoxia and hypoxia) it cannot really be discerned that these results were simply a response to exercise training. No change in C/F ratio was described in the rectus femoris or biceps brachii of climbers regularly engaged into intense climbing, walking, carrying activities at altitudes above 5,250 m (Mizuno et al., 2008; Levett et al., 2012).

Finally, the use of exercise training in hypoxia for improving certain health outcomes (biomechanical limitations, obesity, hypertension, aging) is also gaining a high interest (Burtscher et al., 2004; Wiesner et al., 2010; Verges et al., 2015; Millet et al., 2016; Kong et al., 2017; Pramsohler et al., 2017; Camacho-Cardenosa et al., 2018, 2019, 2020; Ramos-Campo et al., 2019; Jung et al., 2021). In regard to skeletal muscle blood flow, capillarization and angio-adaptation, we and others have shown in rodent models and human patients that exercise interventions could represent a powerful therapeutic avenue to prevent, delay or improve alterations in chronic conditions such as peripheral limb ischemia, diabetes, obesity, chronic obstructive pulmonary diseases (Armstrong et al., 1986; Hudlicka et al., 1994; Gardner and Poehlman, 1995; McDermott et al., 2009; Roudier et al., 2009; Gouzi et al., 2013; Amouzou et al., 2016; Aiken et al., 2019). Yet, to the best of our knowledge the impact of hypoxia training as a therapeutic or preventive approach specifically for conditions affecting muscle capillarization and angio-adaptive activity remains to be investigated.

## Muscle Capillarization, Exercise Training, and Ambient Hypoxia: Key Points

• Prolonged exposure to hypoxia can improve skeletal muscle capillary density likely because of myofiber atrophy. Yet the implication of a true angiogenic response cannot be ruled out.
• Combining training and ambient hypoxia can result in a higher muscle angiogenic response than training alone. Yet whether it could also result in an earlier response remains unknown.
• The existing literature obviously shows non-negligible discrepancy imputable sometimes to a lack of proper controls in early studies and to uncontrolled confounding factors such as activity level and training status, cold exposure, restriction in calory intake, animal growth.

## Skeletal Muscle Capillarization in High-Altitude Native Populations

As mentioned previously, a classical idea is that prolonged exposure of lowlanders to high altitude could result in increased skeletal muscle CD often attributed to myofiber atrophy. Could this represent a phenotypic transition towards highlanders’ muscles (Gilbert-Kawai et al., 2014)?

Interestingly, CD measured in highlanders’ muscles do not seem to differ much from lowlanders. Kayser et al. (1991) have compared the CD from Tibetan Sherpas’ muscles with average CD values from sedentary lowlanders or active climbers before and after a high altitude expedition (Kayser et al., 1991). The average CD was significantly higher (+20%) in Sherpas’ muscles than in sedentary lowlanders’ muscles. However, the training status of the subjects was not taken into consideration. In fact, there was no difference between Sherpas and active climbers before expedition, and a trend for a lower CD (-13%) was even observed in Sherpas’ muscles when compared with post-expedition lowlander climbers’ muscles (Kayser et al., 1991). It is also important to note that the CD values measured in Sherpas’ muscles in this study (Kayser et al., 1991) were compared with CD obtained from different studies (Hoppeler et al., 1990a,b; Oelz et al., 1986). In another study, Kayser et al. (1996) have compared muscles from second-generation Tibetans living at low altitude with Nepalese controls: No difference was observed for muscle CD despite smaller fibers in Tibetans (Kayser et al., 1996).

In an interesting study, Lundby (2004) have compared muscle CD from Aymara subjects, who live permanently around 3,800-4,100 m altitude in Bolivia, with CD values from lowlanders’ muscles before and an 8-weeks sojourn at 4,100 m (Lundby, 2004). Aymara muscle CD was 12% (trend) and 15% (significant) lower than lowlanders’ CD, respectively, measured before and after the altitude sojourn. This apparent lower CD is intriguing given that high-altitude natives had 25-30% smaller myofibers. The C/F ratio was in fact significantly 40% lower in Aymara’s muscles compared to lowlanders.

Altogether, these results suggest that skeletal muscles from high-altitude residents, although presenting smaller fibers, do not have a higher CD than lowlanders’ muscles. The observation of a much lower C/F ratio in high-altitude natives reminds the few studies reporting a tendency for a decreased C/F in lowlanders following a prolonged exposure to hypoxia (Green et al., 1989; Hoppeler et al., 1990a,b; MacDougall et al., 1991; Lundby, 2004). It is then tempting to conclude this section by questioning whether a long-term angio-adaptation of the skeletal muscle to hypoxia could in fact result in some capillary regression. Angio-adaptation ensures an optimal match between the muscle capillarization and the metabolic needs of myofibers. If prolonged hypoxia results in an increased CD and smaller myofibers with decreased mitochondria volume density (Horscroft and Murray, 2014; Favier et al., 2015; Murray and Horscroft, 2016; Horscroft et al., 2017), some existing capillaries might at some point become unnecessary.

## Molecular Aspect of Skeletal Muscle Angio-Adaptive Responses to Hypoxia

**FIGURE 3:**
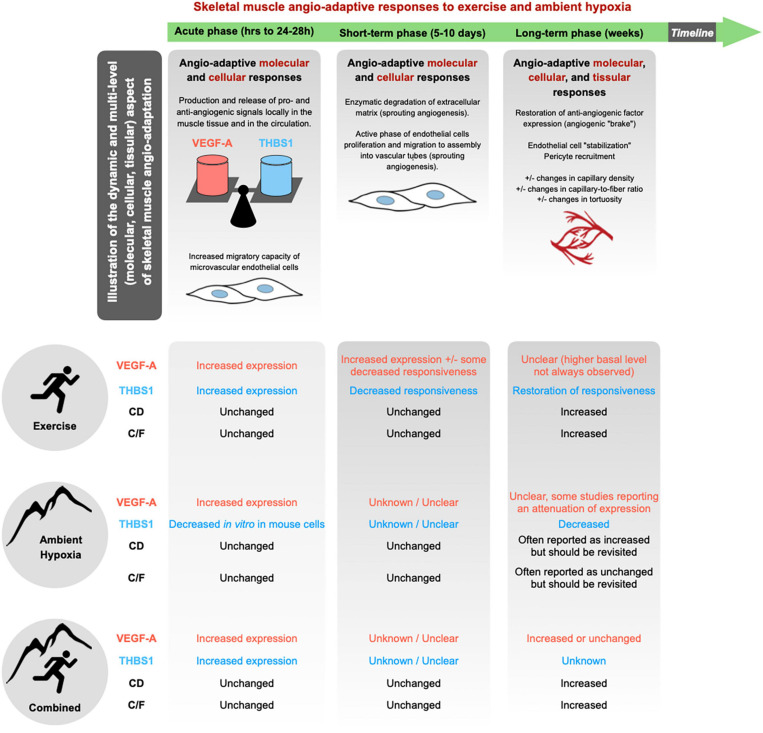
Skeletal muscle angio-adaptation is a complex and dynamic process in which much earlier molecular and cellular events lead to eventual changes observed at the tissular level through modifications in the skeletal muscle’s capillarization. In response to a given stimulus, whether it be the growth, regression, or stabilization of skeletal muscle capillaries, the process is dictated by an intricate balance of pro- and anti-angiogenic factors. Among the plethora of angio-adaptive factors two have been identified as central in the regulation of skeletal muscle capillarization: the pro-angiogenic VEGF-A; and the anti-angiogenic THBS1. Majority of current literature concerning hypoxic muscle angio-adaptation has focused on the regulation of VEGF-A and as such there are current gaps in knowledge concerning the hypoxic regulation of THBS1. Additionally, there is a lack of consensus regarding the impact of ambient hypoxia eliciting changes at the tissular level concerning CD and C/F.

The skeletal muscle capillary network possesses a remarkable plasticity and whether capillaries regress, stabilize or grow in response to a stimulus is largely determined by an intricate balance of pro- and anti-angiogenic molecules (Hoppeler, 1999; Breen et al., 2008; Olfert and Birot, 2011; Egginton and Birot, 2014; Olfert et al., 2015). Among the plethora of angio-adaptive molecules described in the literature, the use of transgenic animal models identified two of them as key regulators of skeletal muscle angio-adaptation: The pro-angiogenic Vascular Endothelial Growth Factor-A (VEGF-A) (Tang et al., 2004; Olfert et al., 2009, 2010; Baum et al., 2017); and the anti-angiogenic thrombospondin-1 (THBS-1) (Malek and Olfert, 2009; Slopack et al., 2014). Different methodological approaches have been used, some targeting VEGF-A in the whole muscle tissue, some targeting it specifically in myofibers (Tang et al., 2004; Breen et al., 2008; Malek and Olfert, 2009; Olfert et al., 2010; Baum et al., 2017). VEGF-A deletion results in: (1) decreased basal level of muscle capillarization, (2) blunted exercise-induced angiogenic response, (3) severely reduced exercise capacity. Targeting the anti-angiogenic THBS-1 has somehow opposite consequences: Better vascularized muscles and greater exercise capacity (Breen et al., 2008; Malek and Olfert, 2009; Olfert et al., 2009, 2015). In the context of the present review on skeletal muscle angio-adaptive responses to hypoxia, it is therefore important to recapitulate the response of VEGF-A and THBS-1 to exercise and ambient altitude (Figure 3).

Most of studies in exercise and altitude physiology have essentially focused on measuring VEGF-A mRNA and protein expression levels. However, how hypoxia can influence VEGF-A expression and activity is in fact very complex (Semenza, 2001; Breen et al., 2008). The *VEGFA* gene can be considered as a hypoxia-sensitive gene since it possesses several Hypoxia Responsive Elements (HRE) recognized by the transcription factor HIF-1. Under hypoxic conditions, the stabilization of HIF-1α and HIF-1 enhanced transcriptional activity lead to increased VEGF-A mRNA levels (Hoppeler et al., 2003). VEGF-A mRNA can then be stabilized via interaction between its 3’ untranslated region and the Human antigen R (HuR protein) (Amadio et al., 2008; Morfoisse et al., 2015; Osera et al., 2015). Interestingly, Tang et al. (2002) have described such interaction between HuR and VEGF-A mRNA in rat ischemic gastrocnemius muscles (Tang et al., 2002). VEGF-A mRNA translation into proteins can also be affected by hypoxia. Indeed, whereas the classical mechanism of mRNA cap-dependent translation can be inhibited under hypoxia, some proteins such as VEGF-A can still be translated by using an alternative translational mechanism where cap-independent translation is initiated via internal ribosome entry sites (IRESs) (Huez et al., 1998; Miller et al., 1998; Stein et al., 1998). The endoplasmic reticulum chaperone Oxygen Regulated Protein-150 (ORP-150) can finally facilitate VEGF-A protein secretion, and an increase in ORP-150 has been reported in rat plantaris muscles following an acute bout of running exercise (Ozawa et al., 2001a,b; Birot et al., 2003). Finally, hypoxia can also stimulate the expression of VEGF-A receptors (Gerber et al., 1997).

A stimulatory effect of exercise on VEGF-A expression has been well described in human and rodent studies. Both mRNA and protein levels are transiently increased after one bout of exercise (Breen et al., 1996, 2008; Gustafsson and Kraus, 2001; Hoppeler and Vogt, 2001; Gustafsson et al., 2002, 2005; Birot et al., 2003; Ameln et al., 2005; Croley et al., 2005; Gavin et al., 2006, 2007; Roudier et al., 2012; Aiken et al., 2016). A study from Breen et al. (1996) has shown that increases in mRNA in rat skeletal muscle were higher and lasted longer when exercise was performed at a higher intensity (Breen et al., 1996). An attenuation of VEGF-A mRNA responsiveness to exercise stimulus has be observed after short-term (few days) training (Gavin and Wagner, 2001; Olfert et al., 2001; Vogt et al., 2001; Kraus et al., 2004).

The effect of acute or chronic exposure to ambient hypoxia itself on VEGF-A expression are less conclusive. Most of the studies that have assessed VEGF-A expression in the muscle tissue have measured mRNA levels only, whereas the protein was essentially quantified in the plasma or serum by ELISA (Asano et al., 1998; Gunga et al., 1999, 2003; Schobersberger et al., 2000; Hanaoka et al., 2003; Oltmanns et al., 2006; Bahtiyar et al., 2007; Dorward et al., 2007; Ding et al., 2012; Morici et al., 2013; Brinkmann et al., 2017; Boos et al., 2018; Kasai et al., 2019; Kasperska and Zembron-Lacny, 2020). Results from the literature remain largely inconsistent, reporting some increase in mRNA or protein (Breen et al., 1996; Gavin et al., 2006), some attenuation (Olfert et al., 2001; Oltmanns et al., 2006; Morici et al., 2013; Boos et al., 2018), or no change (Gunga et al., 2003; Lundby, 2004; Bahtiyar et al., 2007). Additionally, circulating VEGF-A levels measured by ELISA do not necessarily reflect skeletal muscle VEGF-A production.

Finally, some studies have analyzed VEGF-A responsiveness when combining exercise and ambient hypoxia (Breen et al., 1996; Asano et al., 1998; Olfert et al., 2001; Vogt et al., 2001; Gunga et al., 2003; Zoll et al., 2006; Nagahisa et al., 2016; Brocherie et al., 2018; Kasperska and Zembron-Lacny, 2020). Breen et al. (1996) have shown that VEGF-A mRNA expression in rat skeletal muscle was higher and last longer when exercise was performed in ambient hypoxia compared to a normoxic environment (Breen et al., 1996). Brocherie et al. (2018) have compared the effect of different modalities of exercise training combined with hypoxia exposure (Brocherie et al., 2018). VEGF-A mRNA levels were only increased in human skeletal muscle in their model of Live High-Train Low and High (LHTLH) that combined passive exposure to ambient hypoxia and exercise training session at maximal intensity under hypoxia, and no change in VEGF-A was observed in the other two models (Live High-Train Low, LHTL, and Live Low-Train Low, LLTL). This study is in line with previous results reported by Vogt et al. (2001) who analyzed VEGF-A mRNA expression in response to a training program realized either at high or low intensity and either in normoxia or hypoxia (6 weeks at an equivalent of 3,850 m) (Vogt et al., 2001). Whereas training at high intensity in normoxia and training at low intensity in hypoxia only led to a trend for an increase in VEGF-A mRNA (respectively, +13 and +17%), only the combination of training at high intensity in hypoxia resulted in significant increase (+72%). These studies support the idea that combining exercise and ambient hypoxia could exacerbate the angio-adaptive response of the skeletal muscle tissue. If the duration, nature and intensity of the training program are key parameters, all these studies also point out the importance of the duration and intensity of hypoxia exposure. This has recently led to the notion of hypoxic dose (Lundby et al., 2009, 2012; D’Hulst and Deldicque, 2017; Millet et al., 2017; Girard et al., 2020).

Thrombospondin-1 has been identified as a potent anti-angiogenic factor in the skeletal muscle tissue (Malek and Olfert, 2009; Hellsten and Hoier, 2014). Similar to VEGF-A, one bout of exercise stimulates the expression of THBS1mRNA and protein in skeletal muscle (Olfert et al., 2006; Malek and Olfert, 2009; Slopack et al., 2014) whereas short-term training is accompanied by a progressive loss of responsiveness of THBS1 to exercise stimulus (Olfert et al., 2006; Slopack et al., 2014; Hoier et al., 2020; Figure 3). This responsiveness, however, appears to be restored with long-term training (Olfert et al., 2006; Hoier et al., 2012; Slopack et al., 2014). The decreased responsiveness of THBS1 during training could in fact contribute to shifting the skeletal muscle angio-adaptive balance towards its pro-angiogenic side, reflected at the tissue level by a pro-angiogenic microenvironment prone to capillary formation. This has led to the hypothesis that exercise-induced angiogenesis might in fact be more controlled by a decrease in anti-angiogenic factors rather than an increase in pro-angiogenic ones (Olfert and Birot, 2011; Hellsten and Hoier, 2014; Olfert et al., 2015; Olfert, 2016). This could apply to capillary regression, as during detraining or muscle hypokinesia, with an increase expression of anti-angiogenic factors shifting the angio-adaptive balance the opposite way (Roudier et al., 2009, 2010; Kishlyansky et al., 2010; Olfert and Birot, 2011; Olenich et al., 2014).

Long-term training does not seem to alter the basal expression level of THBS1 in rodent and human healthy skeletal muscles (Olfert et al., 2006; Hoier et al., 2012; Gliemann et al., 2015) although Yoshioka et al. (2003) have reported higher THBS1 gene expression in skeletal muscles from endurance athletes compared to sedentary subjects (Yoshioka et al., 2003). Interestingly, Gouzi et al. (2013) have shown that muscle THBS1 protein levels could be reduced in response to prolonged training in patients with chronic obstructive pulmonary disease (Gouzi et al., 2013). This reinforces the idea of using exercise training as a therapeutic avenue for clinical conditions with skeletal muscle microvascular alterations. THBS1 expression was indeed found to be increased in rodent skeletal muscles in the context of diabetes, pre-diabetes and hindlimb ischemia (Kivelä et al., 2006, 2008; Roudier et al., 2013; Dunford et al., 2017; Aiken et al., 2019).

The effect of hypoxia alone on THBS1 expression has provided mixed results essentially from *in vitro* experiments. Phelan et al. (1998) have described an increased expression of THBS1 mRNA and protein in endothelial cells exposed to severe hypoxia (1%O_2_) (Phelan et al., 1998). Conversely, Yadav et al. (2014) have observed a decrease in THBS1 expression in differentiated murine C2C12 myotubes in response to hypoxia (Yadav et al., 2014). Finally, THBS1 expression was found to be increased both in rodent and human ischemic skeletal muscles (Roudier et al., 2013).

To our knowledge, there is very limited data regarding THBS1 expression in skeletal muscle when combining exercise stimulus and ambient hypoxia. Olfert et al. (2006) have analyzed THBS1 mRNA expression in skeletal muscles from rats kept sedentary or enrolled into a 8-weeks endurance running program (Olfert et al., 2006). At the end of the training program, animals were performing one bout of intense running exercise. Data suggests that endurance training did not alter the basal expression level of THBS1. However, chronic hypoxia exposure resulted in lower basal expression level (–44%) as well as THBS-1 expression in response to one bout of exercise (–48%).

As a conclusion to this section, it is important to keep in mind that VEGF-A and THBS1 are only two members of the large family of angio-adaptive molecules susceptible to influence the skeletal muscle angio-adaptive responses to exercise and ambient hypoxia. The expression levels of some other angio-adaptive molecules have been measured in skeletal muscle tissue in the context of exposure to ambient hypoxia, such as basic Fibroblast Growth Factor (bFGF), Transforming Growth Factor-β (TGF-β), VEGF receptors, leptin (Breen et al., 1996; Olfert et al., 2001; Patitucci et al., 2009; Morici et al., 2013). However, these measurements remain very anectodical, and more research in this area is needed.

Here, we have also essentially reviewed VEGF-A and THBS1 expression levels, which do not reflect the functionality of these molecules, their interaction between each other’s and their receptors.

As illustrated in Figure 3, the angio-adaptive response to a given stimulus is a complex and dynamic process that involves molecular, cellular, and tissular responses. There is for example a lack of knowledge regarding cross-talks between muscle cells. For instance, how angio-adaptive signals originating from a contractile myofiber will stimulate neighboring endothelial cells to proliferate and migrate to form new capillaries.

Ideally, evaluating the muscle angio-adaptive response to hypoxia should be integrative. For example, an absence of significant change in capillarization does not rule out any angio-adaptive responses at a cellular or molecular level. Finally, the interpretation of results from the literature is very complex with a certain discrepancy between studies. Such divergence can obviously have several origins: Animal versus human studies; healthy versus pathological conditions; training status; exercise protocols; hypoxia level and duration; confounding environmental stressors (cold, air pollution); time of sample collection; methodology (northern blotting, qPCR, western blotting, ELISA, histochemistry).

## The Skeletal Muscle as an Endocrine Organ and the Role of Angio-Adaptive Myokines

As previously mentioned, many studies studying angiogenic responses to hypoxia and exercise have quantified circulating VEGF-A protein levels by ELISA. Several studies have aimed to link changes in circulating VEGF-A levels with the susceptibility to develop acute or chronic mountain sickness (Tissot van Patot et al., 2005; Dorward et al., 2007; Nilles et al., 2009; Schommer et al., 2011; Espinoza et al., 2014). Regarding THBS1, this anti-angiogenic molecule seems to be involved in the pathophysiology of hypoxia-induced pulmonary hypertension and right ventricular hypertrophy (Ochoa et al., 2010; Bauer et al., 2012; Rogers et al., 2017). Kaiser et al. (2016) have reported elevated serum THBS1 levels and strong correlations of serum THBS1 to mean pulmonary artery pressure and pulmonary vascular resistance in patients suffering from pulmonary hypertension (Kaiser et al., 2016).

Interestingly, many circulating angio-adaptive molecules, either pro- or anti-angiogenic, could therefore represent valuable biomarkers to evaluate for example the response of athletes to a specific training program in hypoxia or the impact of a therapeutic exercise intervention in a group of patients. More research in identifying the patho-physiological relevance of circulating angio-adaptive biomarkers would be exciting and essential.

The quantification of circulating angio-adaptive molecules also points out the role of the skeletal muscle as an endocrine organ secreting myokines to act on distant organs such as bones, brain, fat, and liver (Fabel et al., 2003; Schnyder and Handschin, 2015; Delezie and Handschin, 2018; Kim et al., 2019; Gomarasca et al., 2020). Fabel et al. (2003) have demonstrated that peripherally produced VEGF-A seems necessary for running-induced improvements in hippocampal neurogenesis (Fabel et al., 2003). Rich et al. (2017) confirmed that VEGF-A produced by skeletal myofibers plays an important role in hippocampal neurogenesis (Rich et al., 2017). Interestingly, it was also suggested that VEGF-A meditated neurogenesis could provide a neuroprotective effect and could be essential for attenuating decrements to cognitive function experienced with ambient hypoxia during high altitude exposure (Koester-Hegmann et al., 2019).

## Molecular Aspect of Skeletal Muscle Angio-Adaptive Responses to Hypoxia: Key Points

• Skeletal muscle angio-adaptation is a complex and dynamic process combining molecular and cellular responses that will ultimately alter muscle capillarization.
• Several angio-adaptive molecules have been described in the skeletal muscle tissue in the context of exercise-induced angio-adaptation. Yet, their characterization in the context of exposure to ambient hypoxia remains largely unknown.
• There is a current lack of consensus in the literature largely due to confounding experimental variables regarding the expression of muscle VEGF-A and circulating VEGF-A in response to ambient hypoxia alone and to training in hypoxia.
• Anti-angiogenic molecules, such as THBS1, could in fact impact skeletal muscle capillarization to a greater extent than VEGF-A. Their contribution in skeletal muscle angio-adaptive responses to exercise and hypoxia is however, understudied.
• The patho-physiological relevance of circulating angio-adaptive molecules as biomarkers remains poorly documented.
• The role of the skeletal muscle tissue as an endocrine organ secreting angio-adaptive myokines to act on distant organs in the context of exercise and hypoxia is an exciting research avenue.

## Skeletal Muscle Angio-Adaptive Responses to Hypoxia: Do Normobaric and Hypobaric Conditions Differ?

The research studies discussed in our review were conducted in three types of environments: Field experiments with real altitude exposure versus hypoxic chambers simulating altitude. Chambers where the barometric pressure and partial oxygen pressure are decreased represent a hypobaric environment, closer to the physics of real altitude, whereas normobaric chambers generate a hypoxic environment by decreasing the fraction of oxygen in the inspired air. Whether normobaric and hypobaric hypoxia are equivalent and then interchangeable has been an intense source of scientific debate, particularly with regards to their impact on exercise performance and various physiological parameters. Systematic reviews and “points-counterpoints” discussions have however, not enable researchers to reach any consensus (Girard et al., 2012; Millet et al., 2012a,b, 2013; Mounier and Brugniaux, 2012a,b; Faiss et al., 2013; Debevec and Millet, 2014; Richard et al., 2014; Coppel et al., 2015; DiPasquale et al., 2016; Richalet, 2020a,b). Discrepancy between studies can be attributable to many cofounding factors: Different degrees of hypoxia, themselves determined either by barometric pressure, inspired PO_2_, oxygen fraction; seasonal and geographical differences in barometric pressure; air temperature and humidity; additional environmental stressors such as cold exposure; duration of exposure; presence or not of exercise interventions, and if so different exercise protocols; characteristics of subjects; animal versus human studies.

**FIGURE 4:**
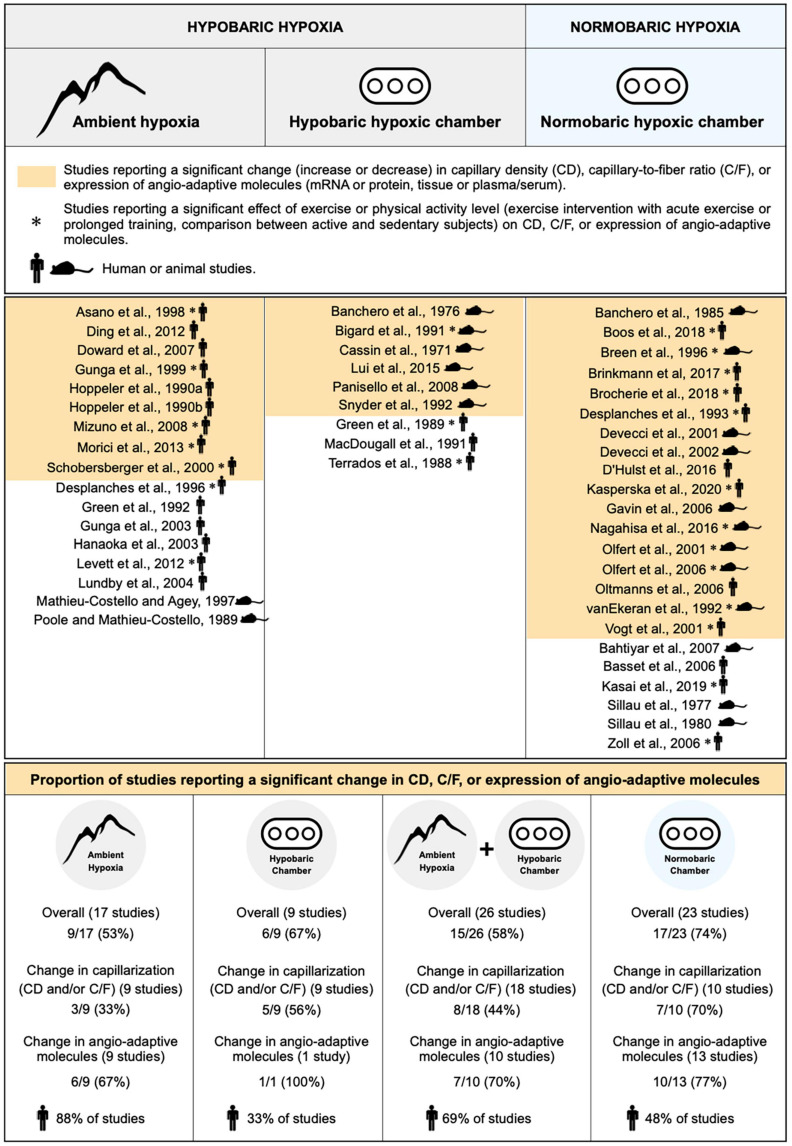
Characterization of the impact of hypobaric and normobaric hypoxia on skeletal muscle angio-adaptive responses. Original research studies cited in the review and analyzing capillary density (CD), capillary-to-fiber ratio (C/F), and expression levels of angio-adaptive molecules in skeletal muscle tissue in response to ambient hypoxia exposure were characterized based on the nature of their hypoxic environment: Hypobaric or normobaric. Studies reporting significant changes in capillarization (CD or C/F), in molecular expression, or in both (“Overall”) are highlighted in color. Studies involving a physical activity component are identified by an asterisk. The mouse and human silhouette symbols distinguish studies conducted in animal models or human subjects.

To the best of our knowledge, whether normobaric and hypobaric hypoxia could differently affect the angio-adaptive responses of the skeletal muscle has never been investigated. We have therefore revisited the original research studies cited in our review that analyzed CD, C/F, or the expression levels of angio-adaptive molecules (mRNA or protein levels, tissue or circulating levels) in response to ambient hypoxia exposure. This represents 49 original studies (Figure 4). We separated them accordingly to the hypoxic environment used: Field studies (hypobaric), hypobaric chambers, combined field and hypobaric chambers (both hypobaric environments), and normobaric chambers. Studies involving physical activity (comparing active versus less active subjects, including one bout of exercise or prolonged training) were considered only if they possessed all required control groups enabling the evaluation of the impact of ambient hypoxia *per se*. We also distinguished studies conducted in animal models or human subjects. Finally, we determined in each category the percentage of studies reporting significant changes (increase or decrease) in capillarization (CD and/or C/F) and expression of angio-adaptive molecules.

Unfortunately, the information presented in Figure 4 does not really help in answering the question on whether normobaric and hypobaric hypoxia could differently affect the angio-adaptive responses of the skeletal muscle. Indeed, when considering only the studies reporting significant changes in capillarization, a distinction could be made between those conducted in normobaric chambers (70% presenting significant changes) versus studies run in a hypobaric environment (33% for field studies, 56% for hypobaric chambers, and 44% for combined hypobaric environments). However, no such distinction could be made when considering only the studies reporting significant changes in angio-adaptive molecules (67% for field studies, 70% for combined hypobaric environments, and 77% for studies conducted in normobaric chambers). Finally, when looking at “overall” changes (capillarization and angio-adaptive molecules), it seems that a larger proportion of studies conducted in hypoxic chambers, whether normobaric or hypobaric, report significant changes (respectively, 74% and 67%) compared to field studies (53%). As mentioned earlier, a possible explanation could be that field studies often present cofounding factors. Another interesting observation from Figure 4 is that these conditionings in hypoxic chambers were mainly performed in animal models whereas field studies were essentially involving human subjects (respectively, 33% and 48% of human studies involving hypobaric and normobaric chambers versus 88% for field studies). Similar durations of exposure to hypoxia will obviously not represent the same proportion of a lifespan between rodents and humans.

Based on this analysis, we do not believe that any strong consensus can be established regarding the impact of hypobaric versus normobaric hypoxia on skeletal muscle angio-adaptive responses.

## Conclusion

**FIGURE 5:**
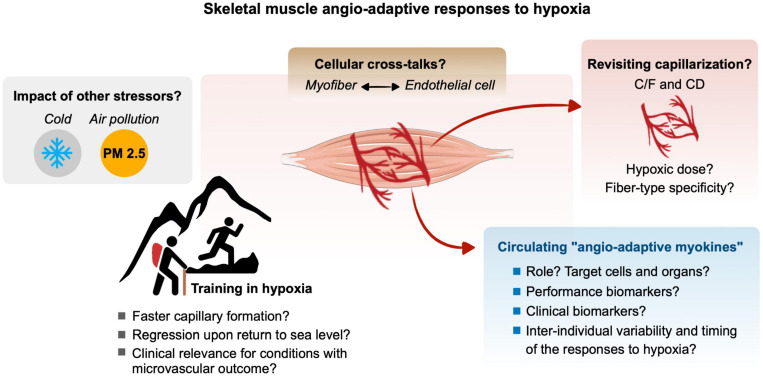
Gap of knowledge and future research directions for a better understanding of the molecular, cellular and tissular angio-adaptive responses of the skeletal muscle tissue to hypoxia, particularly in the context of ambient hypoxia and exercise-induced local hypoxia. Refer to the different text sections for details.

Hypoxia, defined as a reduction of oxygen availability can occur in the skeletal muscle tissue of an individual exposed to ambient hypoxia as well as during physical exercise if the oxygen supply to contracting myofibers cannot match their increased metabolic needs. The superimposition of these two stressors, ambient hypoxia exposure and exercise-induced local hypoxia, can lead to an exacerbation of the hypoxic stress experienced by the skeletal muscle. The capillary-to-myofiber interface serves as the site for the exchange of oxygen, nutrients, metabolic heat, and waste between the blood and myofibers. As such, the capillary microvasculature is tightly related to the functional capacity of the skeletal muscle. The capillary microvasculature is a highly adaptive tissue with remarkably plasticity that can grow or regress to various physiological, pathological, and environmental stressors, a process named angio-adaptation. Skeletal muscle angio-adaptation involves complex and dynamic molecular and cellular responses. Given the relevance of skeletal muscle angio-adaptation in response to hypoxia to mountaineers, athletes, and clinical populations, this review aimed to delineate the existing literature and identify current gaps in the knowledge of this field of environmental and exercise physiology (Figure 5).

## Author Contributions

Both authors PL and OB have equally contributed to the design and writing of the manuscript and its figures.

## Conflict of Interest

The authors declare that the research was conducted in the absence of any commercial or financial relationships that could be construed as a potential conflict of interest.

## Publisher’s Note

All claims expressed in this article are solely those of the authors and do not necessarily represent those of their affiliated organizations, or those of the publisher, the editors and the reviewers. Any product that may be evaluated in this article, or claim that may be made by its manufacturer, is not guaranteed or endorsed by the publisher.
